# Supplementary material for: Ultra-low-current driven InGaN blue micro light-emitting diodes for electrically efficient and self-heating relaxed microdisplay
Source: Nat Commun. 2023 Mar 17;14:1386. doi: 10.1038/s41467-023-36773-w (PMC10023660; doi:10.1038/s41467-023-36773-w)
Supplement: Supplementary file 1 — Supplementary Information [file 41467_2023_36773_MOESM1_ESM.pdf]

Supplementary Information for

Ultra-low-current Driven InGaN Blue Micro Light-emitting Diodes for Electrically Efficient and Self-heating Relaxed Microdisplay

Woo Jin Baek[1], Juhyuk Park[1], Joonsup Shim[1], Bong Ho Kim[1], Seongchong Park[2], Hyun Soo Kim[1], Dae-Myeong Geum[3, 4]*, and Sang Hyeon Kim[1, 3]*

These authors jointly supervised this work: Sang Hyeon Kim and Dae-Myeong Geum

[1] School of Electrical Engineering, Korea Advanced Institute of Science and Technology (KAIST), Daejeon 34141, Republic of Korea

[2] Division of Physical Metrology, Korea Research Institute of Standards and Science, Daejeon 34113, Republic of Korea

[3] Information and Electronics Research Institute, Korea Advanced Institute of Science and Technology (KAIST), Daejeon 34141, Republic of Korea

[4] School of Electronic Engineering, Chungbuk National University, Chungcheongbuhk-do 28644, Republic of Korea

*e-mail: Dae-Myeong Geum (dmgeum@chungbuk.ac.kr), Sang Hyeon Kim (shkim.ee@kaist.ac.kr)

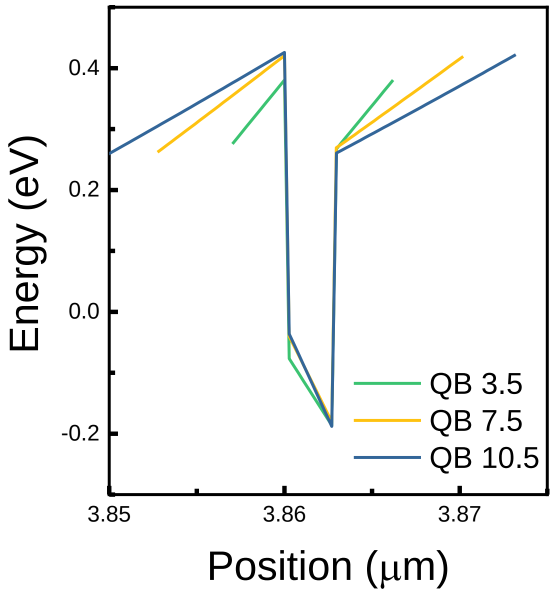

Supplementary Fig. 1 Single conduction band diagram of 2nd QW of QB 3.5, QB 7.5, and QB 10.5 at the current density of 0.1 A/cm^2.

A single conduction band diagram was numerically calculated and compared at 0.1 A/cm^2, and the 2nd quantum-well from the n-doped GaN was aligned in the same position to compare the energy barrier caused by different polarization from QB thickness. The magnitude of the slope indicates the strength of the electric field in the QW and the QB[1], and due to the conservation of the electrostatic field, the net charge is zero, which can be described in following equation[2]:

$$d_{QW}E_{QW} + d_{QB}E_{QB} = 0 \qquad \text{Supplementary Equation (1)}$$

where d_{QW} and d_{QB} are the thickness of QW and QB, and E_{QW} and E_{QB} are the electric fields in QW and QB. When the thickness of QW is fixed, the electric field in the QW increases with increasing QB thickness, which increases the conduction band slope inside the QW. However, as depicted in Supplementary Fig. 1, the energy height of QB increases simultaneously, which is one of the factors that influence the carrier confining capability of the QW.

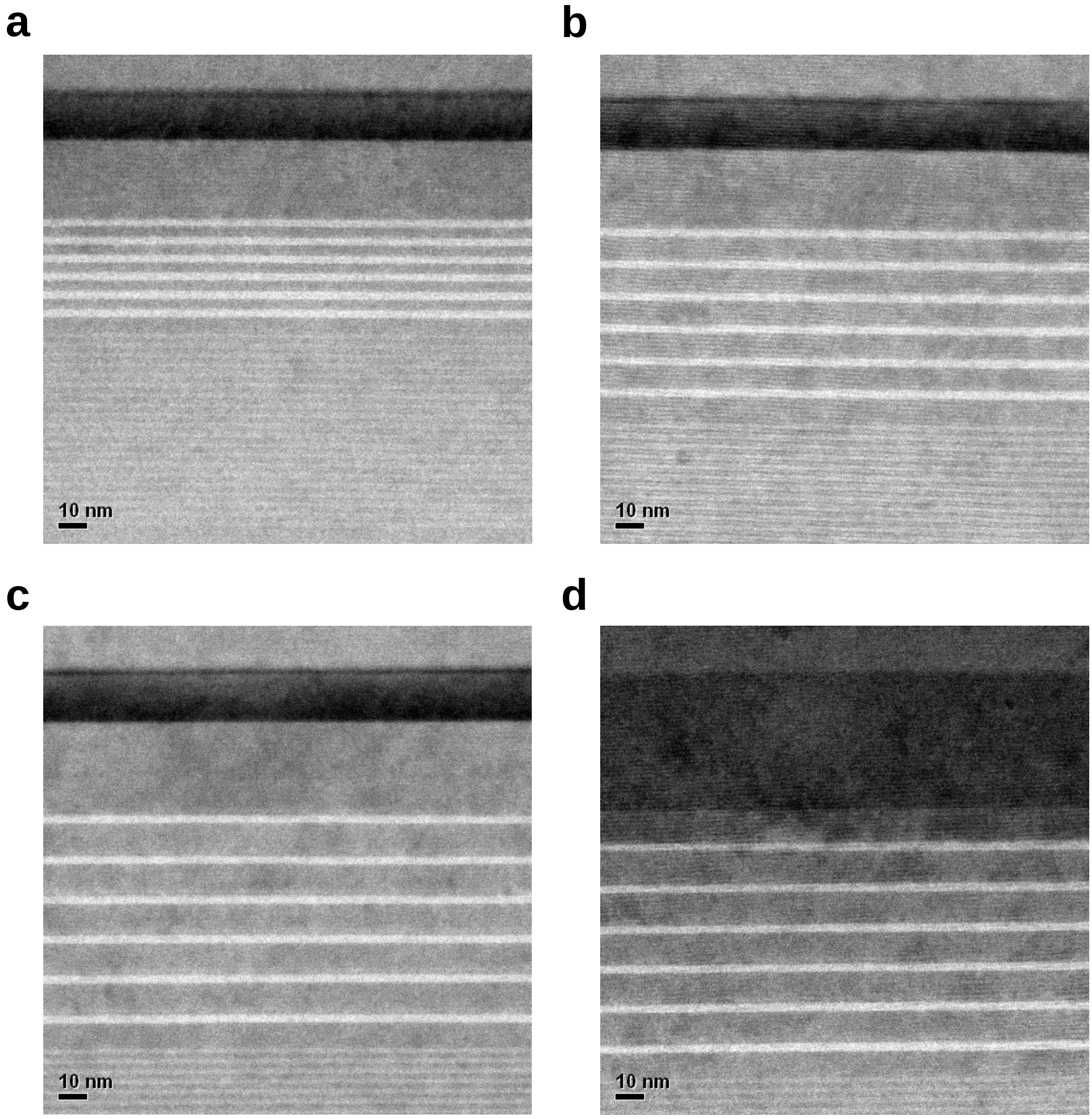

Supplementary Fig. 2 Transmission microscope image of QB 3.5, QB 7.5, QB 10.5, and QB 10.5 Balanced EBL from the superlattice layers to the p-GaN layer. a QB 3.5 **b** QB 7.5 **c** QB 10.5 **d** QB 10.5 Balanced EBL.

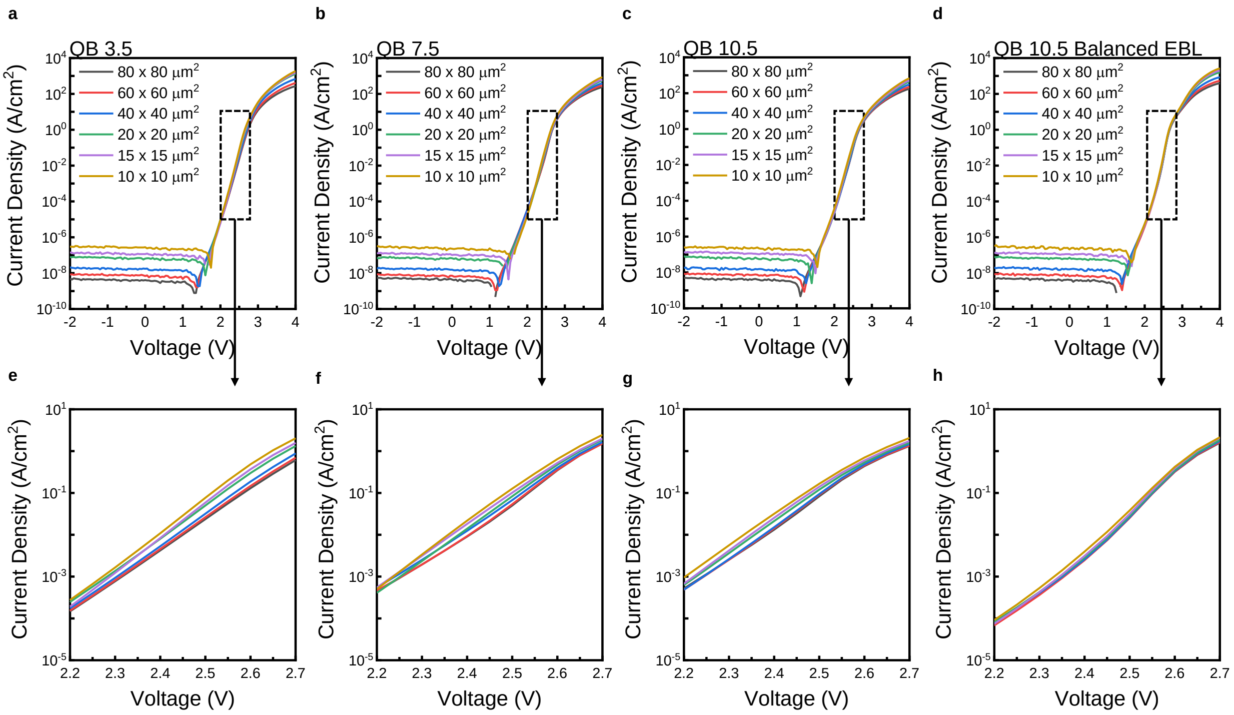

Supplementary Fig. 3 Logarithmic current density versus voltage characteristics of different sizes of μLEDs without sidewall passivation. a&e QB 3.5. **b&f** QB 7.5. **c&g** QB 10.5. **d&h** QB 10.5 Balanced EBL.

Supplementary Fig. 3 shows the *J-V* characteristics of differently sized μLEDs from 80 × 80 μm^2 to 10 × 10 μm^2. The current density in the low forward bias range between 1.5 V and 2.7 V seems overlapping when plotting the *J-V* characteristics of different sizes. However, a gradual increase of current density was observed with decreasing size of the μLEDs when the current density was magnified, and was found the increase rate of QB 3.5 was higher than that of QB 7.5, and QB 7.5 was slightly higher. Specifically, in the case of QB 3.5, the current density at the bias of 2.4 V increase 2468.7 % when the pitch size decreased from 80 × 80 μm^2 to 10 × 10 μm^2, while 140. 6%,139.9%, and 37.1% increase in current density was observed in the case of QB 7.5, QB 10.5, and QB 10.5 Balanced EBL, respectively.

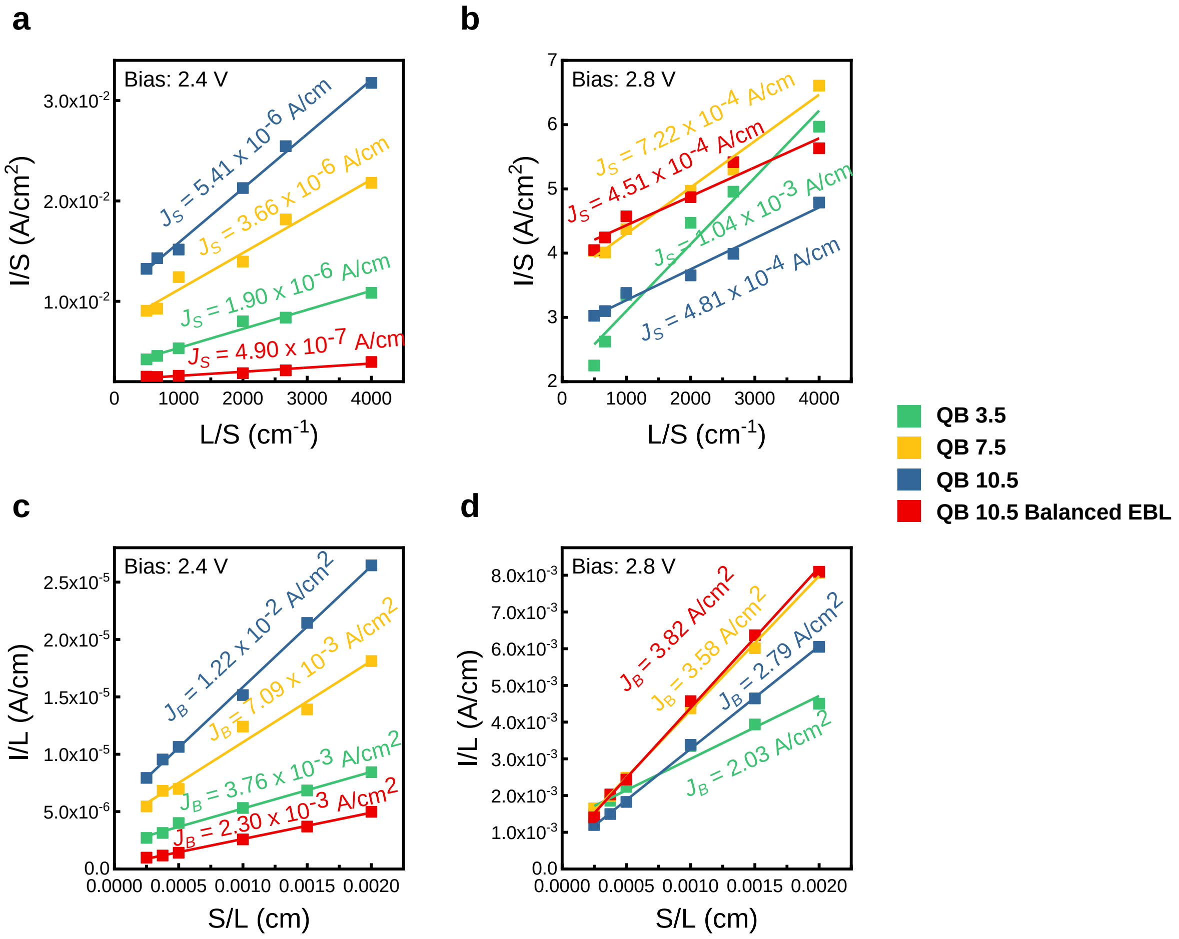

Supplementary Fig. 4 J_S and J_B linear fitting results of QB 3.5, QB 7.5, QB 10.5, and QB 10.5 Balanced EBL. a-b J_S fitting results at forward bias of **a** 2.4 V and **b** 2.8 V. **c-d** J_B fitting results at forward bias of **c** 2.4 V and **d** 2.8 V.

$$I = J_B \times S + J_S \times L. \qquad \text{Supplementary Equation (2)}$$

The supplementary equation (2) describes two possible pathways of forward current in a mesa-shaped diode which is bulk forward current per junction area (J_B) pathing through the mesa and surface current (J_S) pathing around the mesa, where I, S, and L stands for total current, junction area and perimeter of μLEDs mesa[3]. The equation can be transformed into two different equations which ~~is~~ are supplementary equation (3) and (4).

$$\frac{I}{S} = J_S \times \frac{L}{S} + J_B. \qquad \text{Supplementary Equation (3)}$$

$$\frac{I}{L} = J_B \times \frac{S}{L} + J_S. \qquad \text{Supplementary Equation (4)}$$

By plotting the J-V data of differently sized μLED devices from Supplementary Fig. 3 in the form of I/S as the vertical axis and L/S as the horizontal axis, J_S can be extracted by linear fitting the data points, and J_B can be extracted using the same method. As an example, Supplementary Fig. 4 shows the linear fitting results of J_S and J_B when the forward bias is at 2.4 V and 2.8 V.

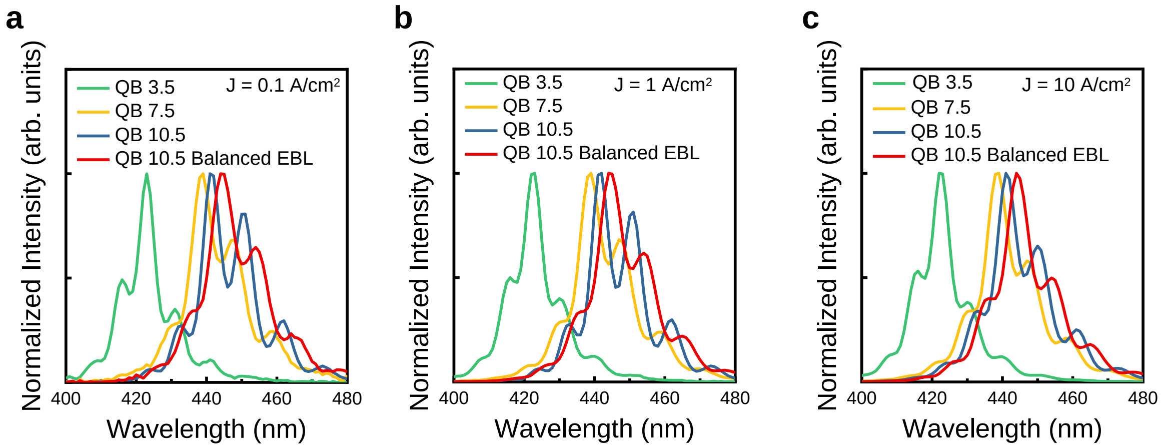

Supplementary Fig. 5 Normalized electroluminescence spectra of devices fabricated from QB 3.5, QB 7.5, QB 10.5, and QB 10.5 Balanced EBL. The operating current density of the device is at **a** 0.1 A/cm^2, **b** 1 A/cm^2, and **c** 10 A/cm^2. The device sizes were 80 × 80 μm^2.

Supplementary Fig. 5 shows the normalized electroluminescence (EL) spectra of devices fabricated from QB 3.5 to QB 10.5 Balanced EBL at different current densities. The EL wavelength trend is similar to the photoluminescence (PL) spectra, where QB 3.5 having a lower peak wavelength than QB 7.5, QB 7.5 lower than QB 10.5, and QB 10.5 similar to QB 10.5 Balanced EBL. A strong Fabry-Perot interference can be observed in the EL spectra due to the large difference in the refractive index of Si substrate, GaN and air[4]. For this reason, we have extracted the peak wavelength and full width at half maximum (FWHM) in Fig. 2**e** and Fig. 4**e** after the Gaussian fitting of the EL spectra.

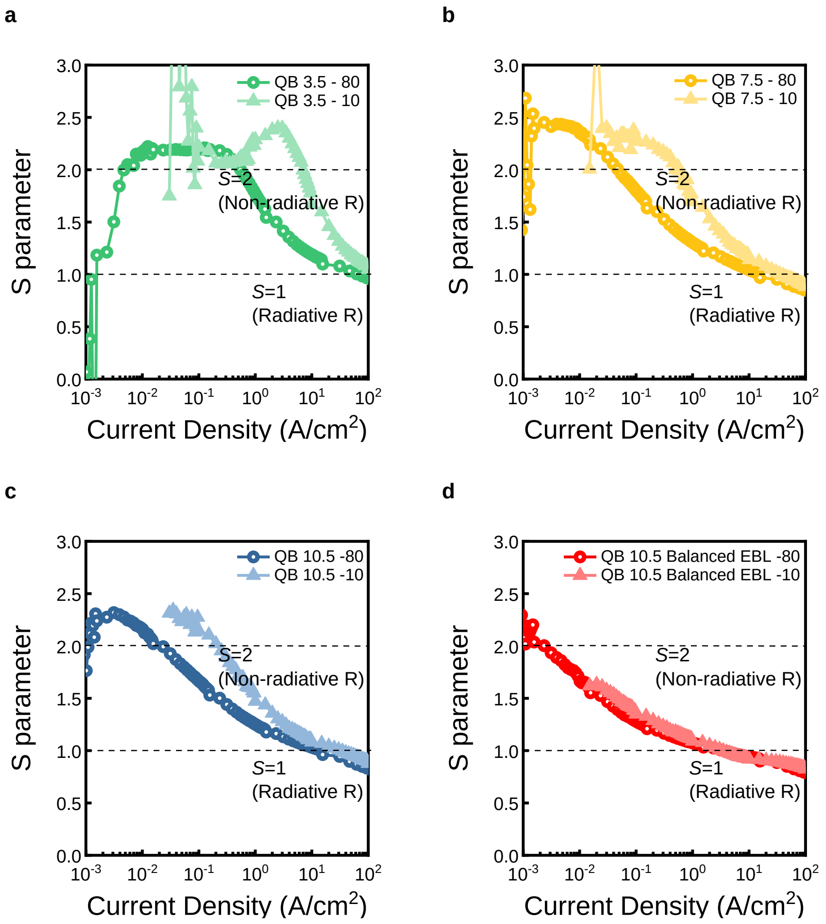

Supplementary Fig. 6 *S* parameter of devices with size of 80 × 80 μm² (80) and 10 × 10 μm² (10). a QB 3.5. **b** QB 7.5. **c** QB10.5. **d** QB 10.5 Balanced EBL.

Supplementary Fig. 6 shows the *S* parameter of 80 × 80 μm^2 and 10 × 10 μm^2 sized μLEDs of QB 3.5, QB 7.5, QB 10.5, and QB 10.5 Balanced EBL. From Fig. 2**f** and Fig. 4**d** in the main paper, it is shown that increasing QB thickness and modifying EBL structure can decrease the current density where the *S* parameter value is below 2 in 80 × 80 μm^2 sized μLEDs. In the case of 10 × 10 μm^2, QB 10.5 Balanced EBL showed a neglectable small shift in the *S* parameter compared to 80 × 80 μm^2 sized device, while significant changes were observed in QB 3.5, QB 7.5, and QB 10.5. However, the degree of shift of the *S* parameter was different in different QB thicknesses. We measured the current density where *S* parameter starts to be lower than 2 at different sizes: QB 3.5 shifted from 0.625 A/cm^2 to 7 A/cm^2, QB 7.5 shifted from 0.0547 A/cm^2 to 0.6 A/cm^2, and QB 10.5 shifted from 0.0234 A/cm^2 to 0.25 A/cm^2. The difference in the shift

of the *S* parameter of QB thickness-controlled samples is corresponding to the different carrier confinement capabilities caused by the tunneling rate. If the tunneling rate is high, the increased lateral diffusion of bypassed carriers can cause more surface recombination in the mesa sidewall, which hinders the efficient injection of carriers to the active region. However, with an efficient injection of holes which can be achieved by modification of EBL described in the main article, the increased radiative recombination reduces the leakage current by emitting carriers as a form of a photon, and if the leakage current is reduced, the holes can be much efficiently injected to the active region since it does not involve in surface recombination.

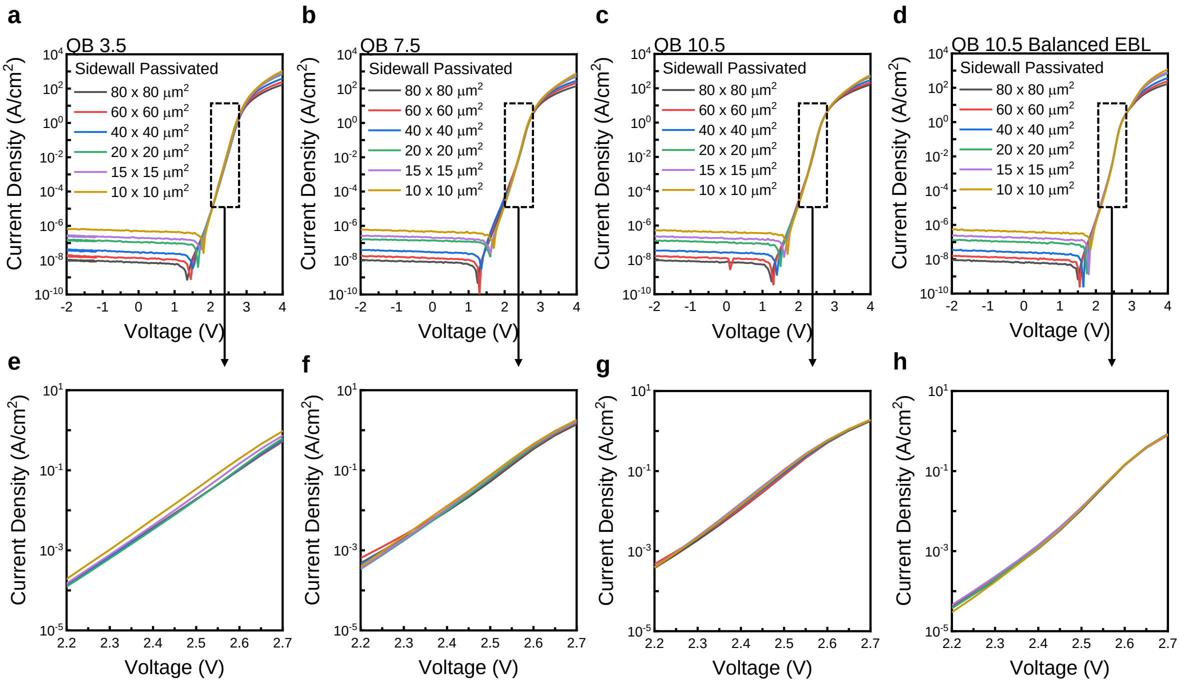

Supplementary Fig. 7 Logarithmic current density versus voltage characteristics of different sizes of μLEDs with sidewall passivation. a&e QB 3.5. **b&f** QB 7.5. **c&g** QB 10.5. **d&h** QB 10.5 Balanced EBL.

In order to observe the impact of sidewall passivation on the surface current, we fabricated the devices with the sidewall passivation scheme. Supplementary Fig. 7 shows the *J-V* characteristics of differently sized μLEDs with sidewall passivation. Comparing the *J-V* characteristics with Supplementary Fig. 3 which shows the results from asdep devices, the gradual increase of current density was reduced dramatically as shown in Supplementary Fig. 7**e-f**. The J_S-V curve and J_S/J_B-V curve are shown in Supplementary Fig. 8 to compare the surface current. We observe a decrease in both J_S and J_S/J_B as shown in Supplementary Fig. 8**a-b.** It is worth mentioning that the trend of decrease of J_S/J_B when the QB thickness increases is still valid even though the sidewall passivation has been conducted, thus the trend in QB 10.5 and QB 10.5 Balanced EBL. Supplementary Fig. 8**c-f** shows the *S* parameter of the asdep and sidewall passivated device with a size of 10 × 10 μm^2. From the figure, a decrease in the *S* parameter was observed especially in the low current density for all structures. However, while the *S* parameters for thin QB thickness have more a dramatic decrease such as QB 3.5, a smaller

change occurred in QB 10.5 Balanced EBL, emphasizing that QB 10.5 Balanced EBL has more immunity to the sidewall efficiency degradation due to its epitaxy nature of less sidewall current.

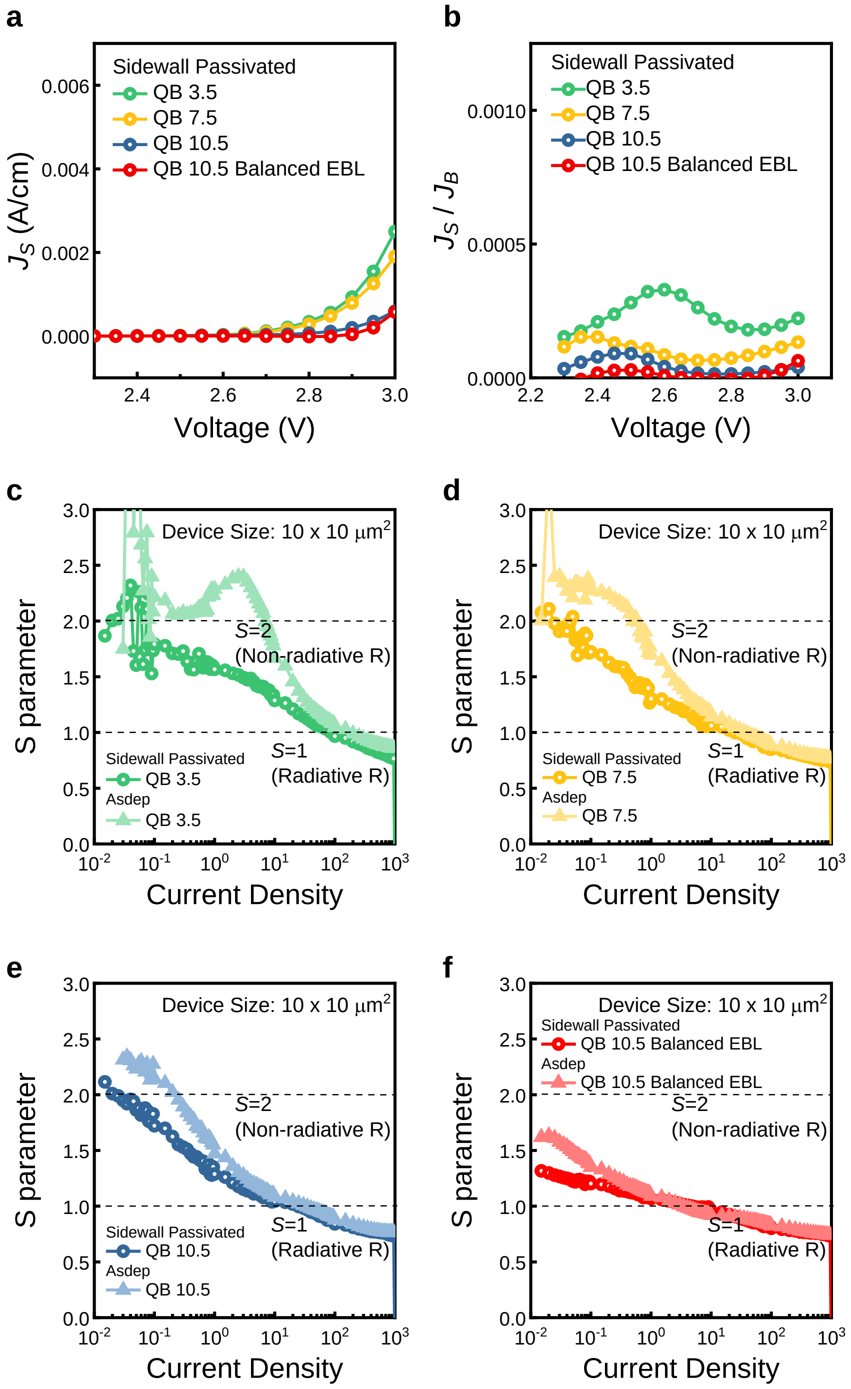

Supplementary Fig. 8 *J_S and J_S/J_B* curve as a function of voltage for the sidewall passivated μLEDs devices. a J_S-V curve **b** J_S/J_B-V curve for QB 3.5 to QB 10.5 Balanced EBL. **c-f** S parameter for the asdep and sidewall passivated device with size of 10 × 10 μm^2 **c** QB 3.5 **d** QB 7.5 **e** QB 10.5 **f** QB 10.5 Balanced EBL.

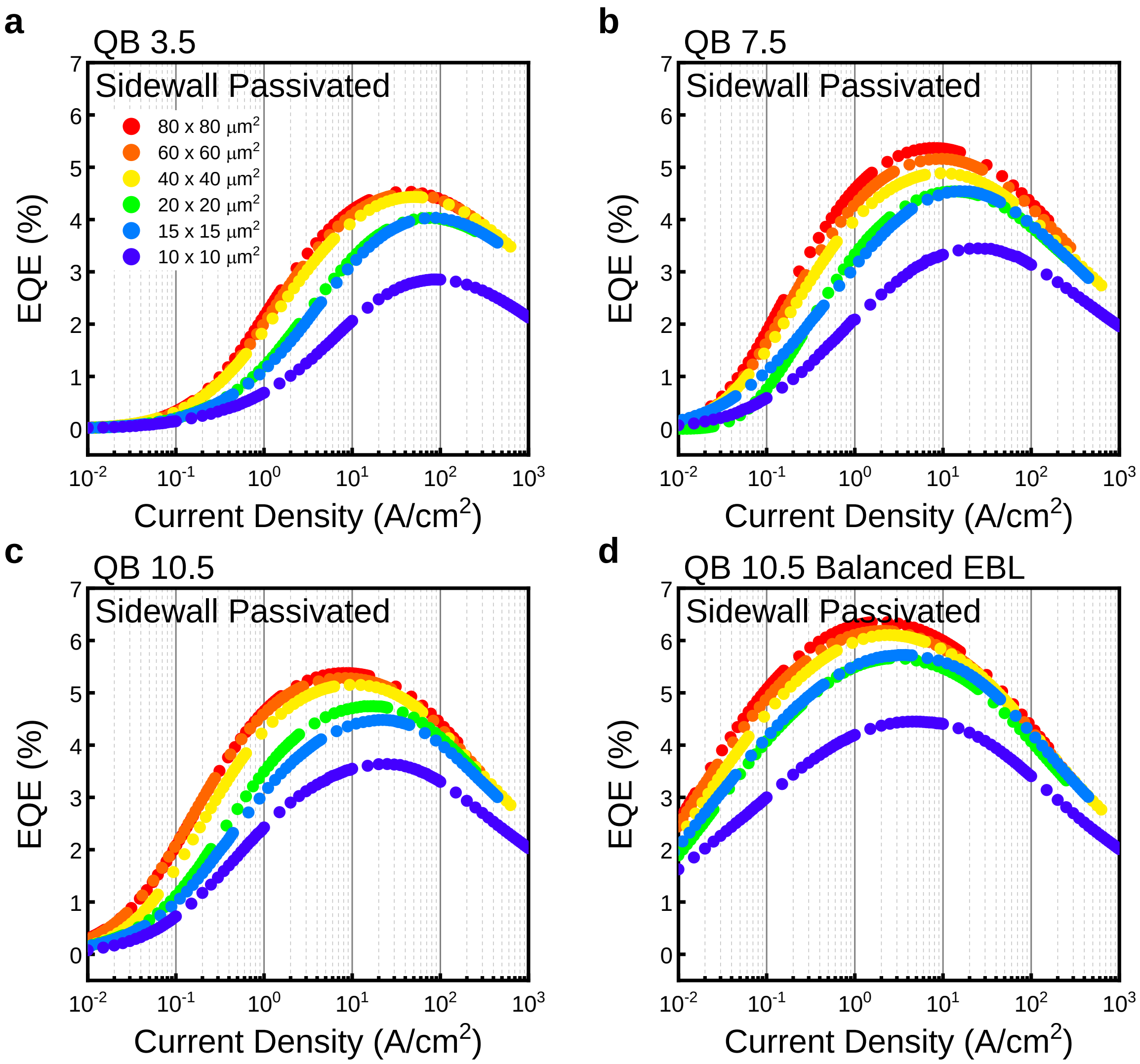

Supplementary Fig. 9 EQE-logarithmic current density curve for sidewall passivated QB 3.5, QB 7.5, QB 10.5 and QB 10.5 Balanced EBL with pitch size from 80 × 80 μm^2 to 10 × 10 μm^2. a QB 3.5 **b** QB 7.5 **c** QB 10.5 **d** QB 10.5 Balanced EBL.

Supplementary Fig. 9 shows the EQE-logarithmic current density curve for the sidewall passivated devices of QB 3.5, QB 7.5, QB 10.5, and QB 10.5 Balanced EBL, and it is summarized and compared with the devices without sidewall passivation in Supplementary Fig. 10. From the figure, we observed enhancements both in maximum EQE and $J_{max\ EQE}$. This is because of the decrease in the surface current as shown in Supplementary Fig. 8. Furthermore, the increasing EQE trend of the epitaxial structure from QB 3.5 to QB 10.5 Balanced EBL is also still valid after the sidewall passivation, which further convinces that the optimization of

epitaxial structure is not an option, but essentials for μLEDs display.

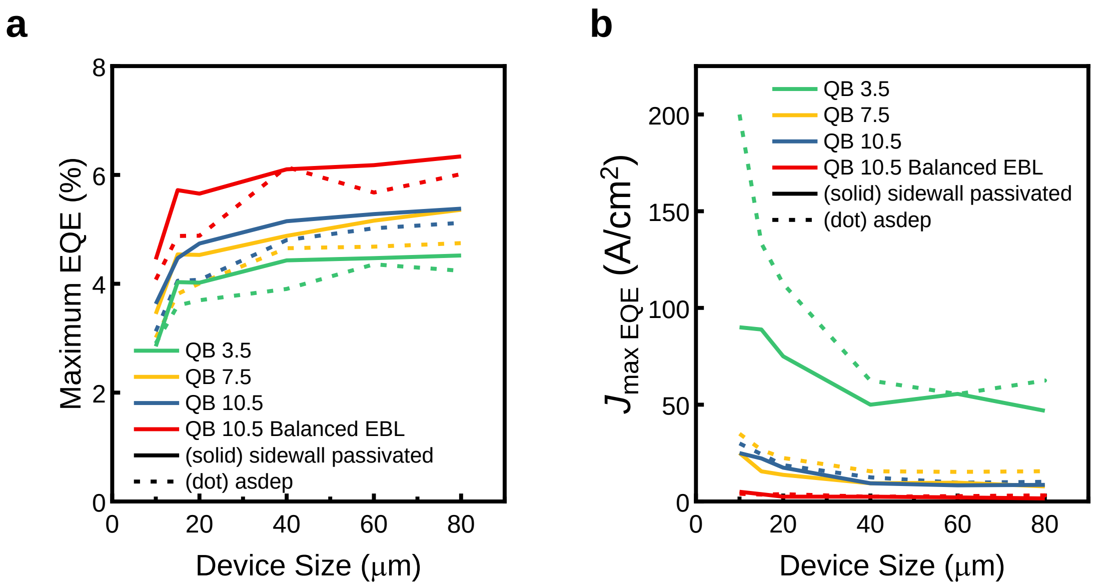

Supplementary Fig. 10 Device performance comparison between the asdep and sidewall passivated device. a maximum EQE and **b** $J_{max\ EQE}$ of asdep and sidewall passivated devices with different sizes.

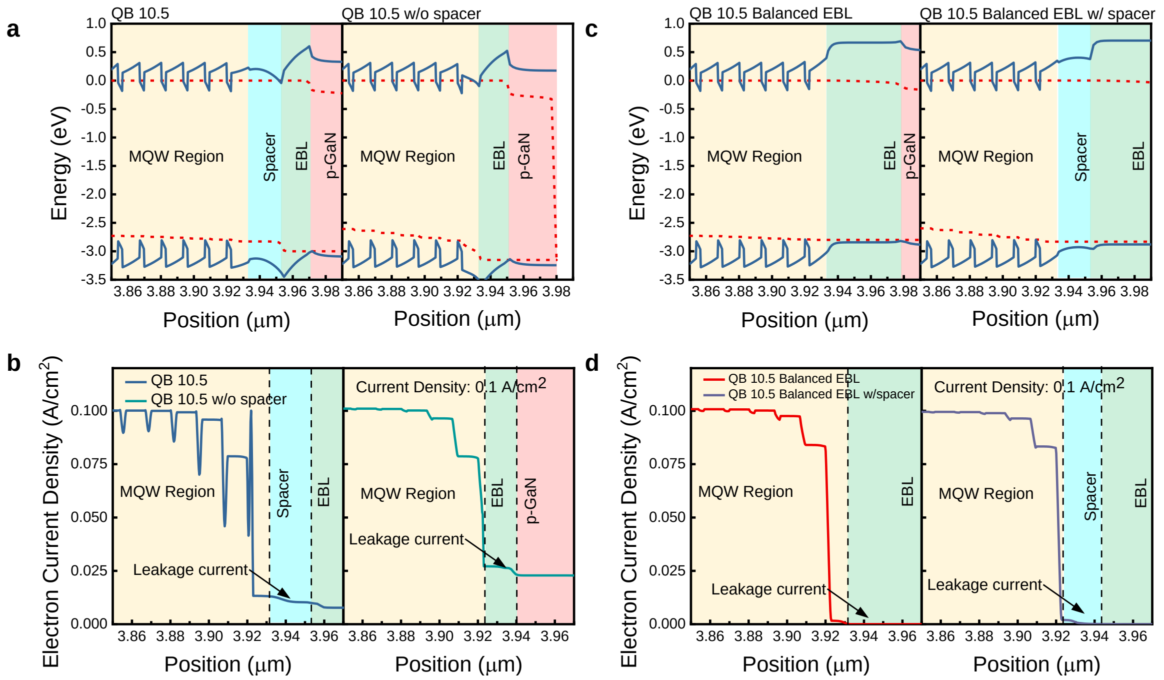

Supplementary Fig. 11 Simulation results of QB 10.5, QB 10.5 w/o spacer, QB 10.5 Balanced EBL and QB 10.5 Balance EBL w/spacer. a,c energy band diagram **b,d** electron current density. The simulations are at the current density of 0.1 A/cm^2.

In order to show the role of the spacer and the reason why no spacer exists in QB 10.5 Balanced EBL, simulations of two additional structure is conducted:

1. QB 10.5 w/o spacer, where the structure is identical to QB 10.5 but without the spacer.

2. QB 10.5 Balanced EBL w/ spacer, where the structure is identical to QB 10.5 Balanced EBL, but there is a spacer layer between the last QB and EBL.

The existence of a spacer is important in QB 3.5, QB 7.5, and QB 10.5. It acts as a layer that compensates the electric field caused by the piezoelectric difference between the last quantum barrier and the electron blocking layer which has a high aluminum composition ($Al_{0.2}In_{0.02}Ga_{0.78}N$). Comparing the energy band diagram of QB 10.5 and QB 10.5 w/o spacer, due to the piezoelectric difference and limited doping concentration caused by high Al composition, a deeper energy dip is formed in the interface between the last QB and EBL in the case of QB 10.5 w/o spacer as shown in Supplementary Fig. 11**a**. As a result, the leakage

current of QB 10.5 w/o spacer is higher as depicted in Supplementary Fig. 11**b**. This increased accumulation of electrons and leakage current can increase the lateral diffusion of electrons to the sidewall in the low current region.

However, through the simulation, we found that the importance of the spacer in QB 10.5 Balanced EBL is not as significant as in QB 10.5. Supplementary Fig. 11**c** shows the energy band diagram QB 10.5 Balanced EBL and QB 10.5 Balanced EBL w/ spacer. Due to the lowered Al composition and induced higher p-type doping concentration of EBL in QB 10.5 Balanced EBL, the energy dip is not formed in the interface between the last QB and EBL in the energy band diagram, which means the necessity to compensate the electric field between this interface is not significant. The simulated leakage current results in Supplementary Fig. 11**d** also show that there is no significant difference between the leakage current.

Supplementary Table 1. Summarizing table of various state-of-art devices and devices in this work. The EQE is compared at the current density of 0.1, 1, 10 A/cm^2. Maximum EQE and $J_{max\ EQE}$ are also shown in the table. (Ley et al.[5], Smith et al.[6], Wong et al.[7], Olivier et al.[8], Sheen et al.[9])

Reference	Device Size (diameter or length)	Device Shape	Substrate	EQE at 0.1 A/cm^2	EQE at 1 A/cm^2	EQE at 10 A/cm^2	Maximum EQE	$J_{max\ EQE}$
Ley et al. (smallest device size)	2 μm	Circular	C-plane Sapphire	1.6%	8.2%	12.7%	13.3%	12 A/cm^2
Ley et al. (Similar size to our smallest device)	10 μm	Circular	C-plane Sapphire	0.25%	5.8%	9.2%	9.2%	13.8 A/cm^2
Smith et al. (smallest device size)	1 μm	Circular	Sapphire	N/A	0.6%	1.2%	2.4%	767 A/cm^2
Smith et al. (Similar size to our smallest device)	10 μm	Circular	Sapphire	0.6%	2.7%	5.7%	6.0 %	32.1 A/cm^2
Wong et al. (smallest device size)	10 μm	Rectangular	PSS	N/A	N/A	21.0%	23.7%	15.1 A/cm^2
Olivier et al. (smallest device size)	10 μm	Rectangular	C-plane Sapphire	N/A	0.3%	4.7%	5.2%	31.5 A/cm^2
Sheen et al. (smallest device size)	530 nm	Circular	Grown on c-plane sapphire but released	N/A	18.8%	21.0%	21.0%	10.2 A/cm^2
This work (asdep)	10 μm	Rectangular	Si (111)	2.08%	3.73%	3.93%	4.08%	4 A/cm^2
This work (sidewall passivated)	10 μm	Rectangular	Si (111)	3.00%	4.19%	4.40%	4.45%	5 A/cm^2

Supplementary Table 2. Thermal profile of μLEDs with a pitch size of 80 × 80 μm^2 and 10 × 10 μm^2 when the light output power per area is 0.1 W/cm^2 and 1 W/cm^2 for asdep and sidewall passivated devices of QB 3.5, QB 7.5, QB 10.5, and QB 10.5 Balanced EBL (BE) samples. The current density above the TRM images shows the corresponding current density. The scale bar is valid only within the μLEDs mesa region since the thermoreflectance coefficient calibration was conducted for the mesa region.

Light Output Power per area	QB 3.5		QB 7.5		QB 10.5		QB 10.5 Balanced EBL	
	Asdep	KOH+ALD	Asdep	KOH+ALD	Asdep	KOH+ALD	Asdep	KOH+ALD
Pitch size: 10 × 10 μm^2								
0.1 W/cm^2	6.05 A/cm^2 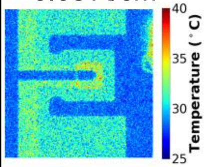	2.81 A/cm^2 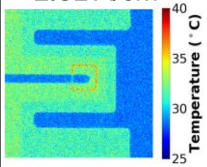	1.82 A/cm^2 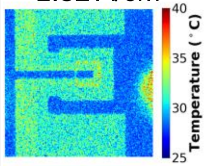	1.52 A/cm^2 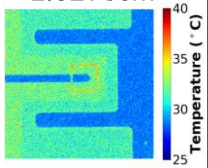	1.57 A/cm^2 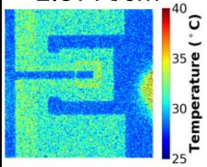	1.36 A/cm^2 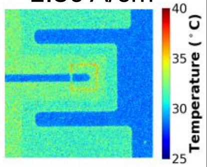	1.18 A/cm^2 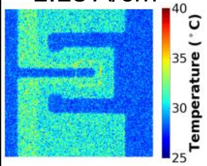	0.86 A/cm^2 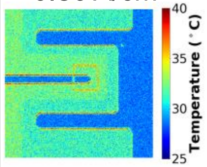
1 W/cm^2	22.70 A/cm^2 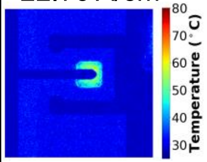	14.81 A/cm^2 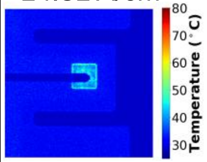	13.29 A/cm^2 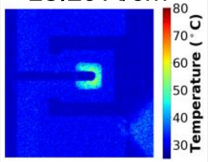	10.74 A/cm^2 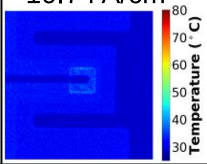	12.48 A/cm^2 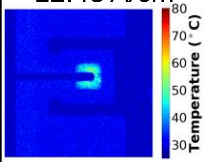	10.13 A/cm^2 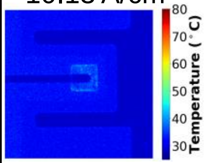	11.04 A/cm^2 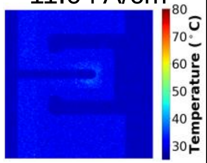	8.13 A/cm^2 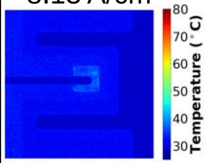
Pitch size: 80 × 80 μm^2								
0.1 W/cm^2	2.34 A/cm^2 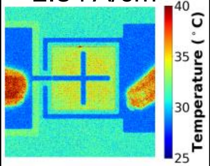	1.41 A/cm^2 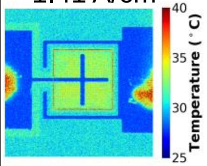	1.01 A/cm^2 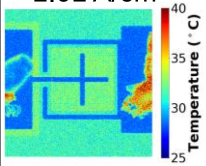	0.85 A/cm^2 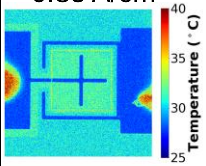	0.86 A/cm^2 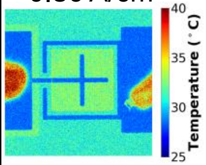	0.80 A/cm^2 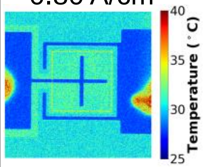	0.70 A/cm^2 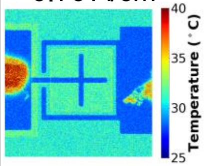	0.58 A/cm^2 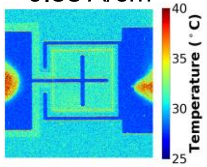
1 W/cm^2	10.16 A/cm^2 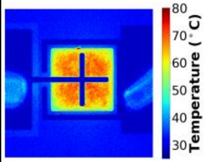	8.59 A/cm^2 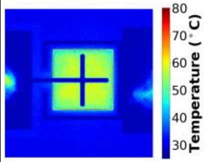	7.81 A/cm^2 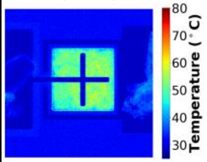	6.73 A/cm^2 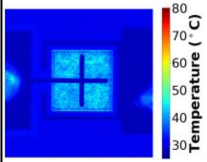	7.03 A/cm^2 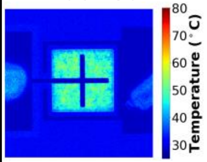	6.72 A/cm^2 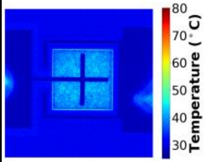	6.25 A/cm^2 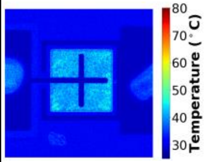	5.83 A/cm^2 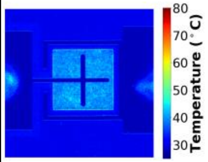

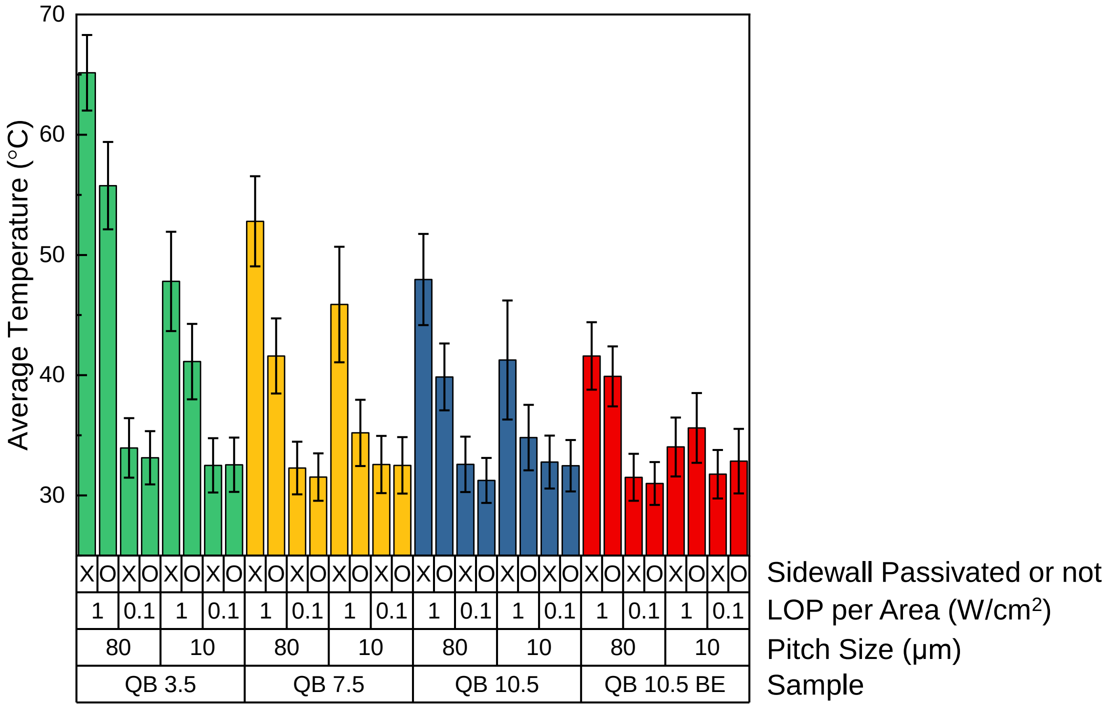

Supplementary Fig. 12 Average temperature of devices fabricated from QB 3.5, QB 7.5, QB 10.5, and QB 10.5 Balanced EBL (BE) with and without sidewall passivation at different sizes and LOP per area. The X corresponds to devices without passivation and O corresponds to devices with sidewall passivation. The pitch size presented in the figure showing 80 μm and 10 μm corresponds to 80 × 80 μm^2 and 10 × 10 μm^2, respectively.

Supplementary Table 2 shows the thermoreflectance microscopy image of QB 3.5 to QB 10.5 Balanced EBL both for devices with and without sidewall passivation with different light output power (LOP) per area at different pitch sizes of 10 μm^2 × 10 μm^2 and 80 μm^2 × 80 μm^2. The resulting average temperature is summarized in Supplementary Fig. 12. The resulting average temperature shows a trend that the sidewall passivated devices with lower average temperature than the devices without sidewall passivation both for 10 μm^2 × 10 μm^2 and 80 μm^2 × 80 μm^2 sized devices, especially at higher LOP per area of 1 W/cm^2. In the meanwhile, at a lower LOP per area of 0.1 W/cm^2, the average temperature has negligible change after the sidewall passivation, which emphasizes the importance of the necessity of low-current operation in μLEDs display.

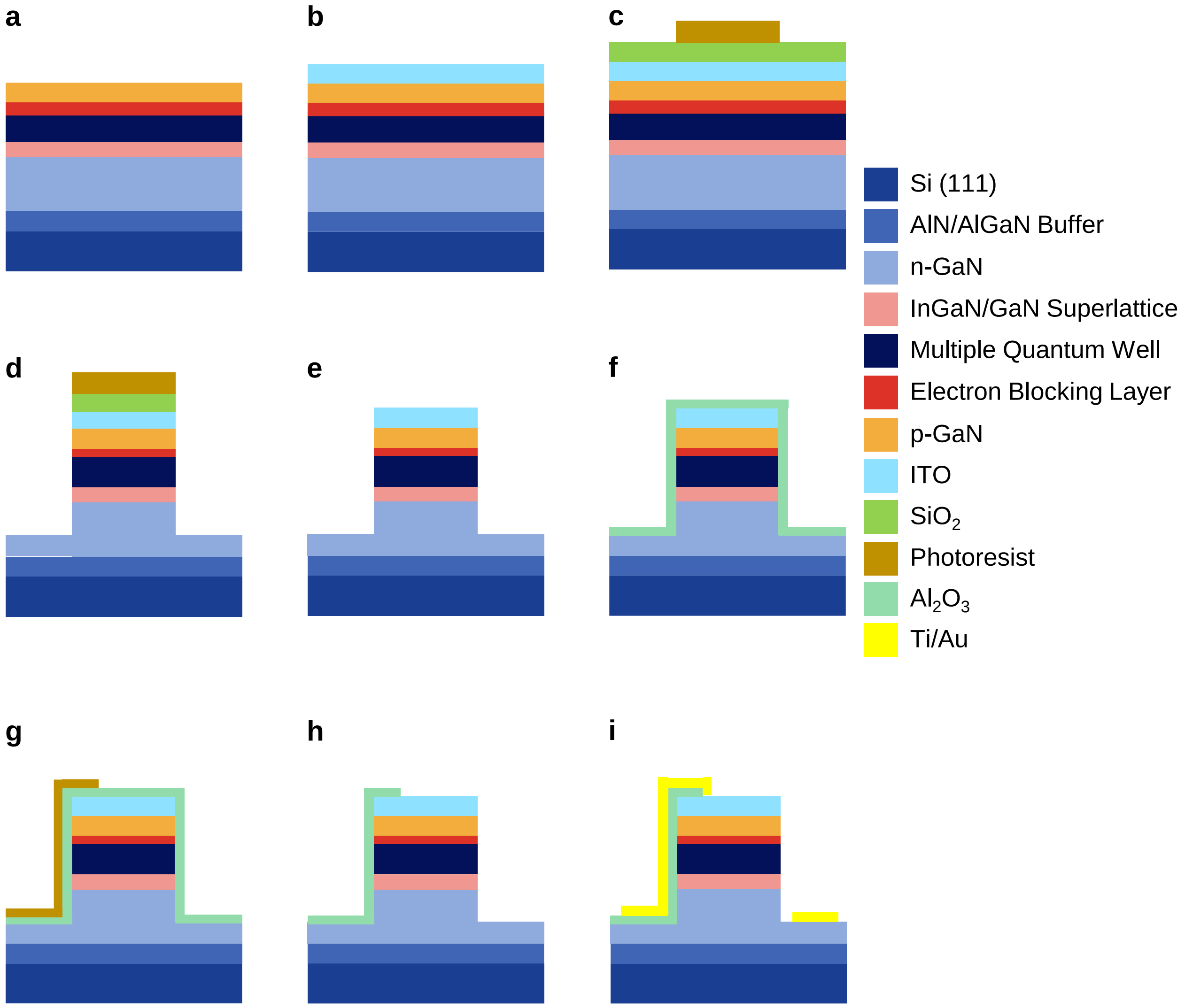

Supplementary Fig. 13 Fabrication process of µLEDs. **a** as-dep condition after acetone, methanol, and iso-propanol cleaning. **b** deposition of ITO. **c** hard masking with PECVD SiO_2 and mesa patterning. **d** defining pixel with ICP-RIE process. **e** removal of PR and SiO_2 mask. **f** deposition of Al_2O_3 for p-type contact isolation. **g** patterning to etching unnecessary parts of Al_2O_3. **h** etching of Al_2O_3. **i** metal deposition for p-type and n-type contact.

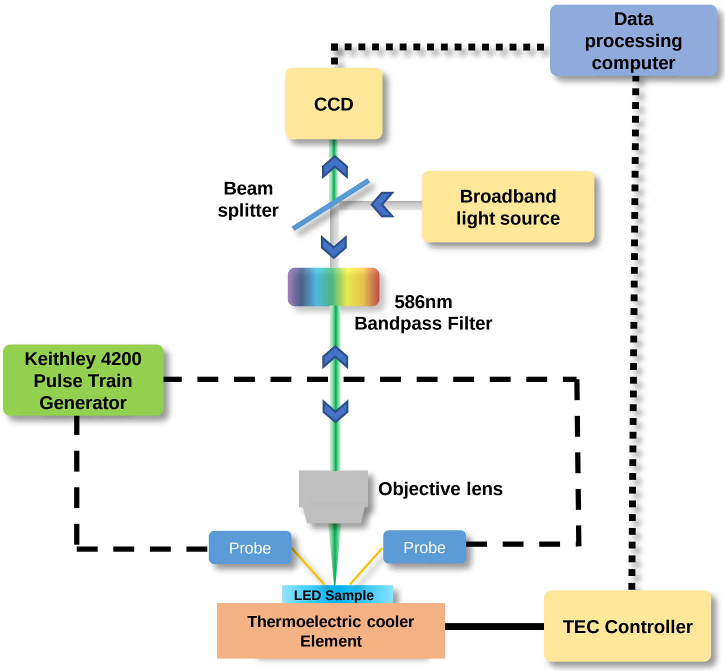

Supplementary Fig. 14 Schematic of measurement setup of thermoreflectance microscopy. The thermoreflectance microscopy (TRM) setup schematic is shown in Supplementary Fig. 14. The broadband light source is passed through a bandpass filter which eliminates the emission wavelength of LED samples. The light is then reflected from the LED sample, and detected by the CCD. Since reflectivity is related to refractive index, and refractive index is influenced by temperature, it is possible to measure the reflectivity change of the sample surface in terms of temperature by controlling the temperature using a thermoelectric cooler element. Supplementary equation (5) describes the approximated relation between the change of reflectivity and the change of temperature in first-order equation[10].

$$\frac{\Delta R}{R} = \left(\frac{1}{R}\frac{dR}{dT}\right)\Delta T = \kappa \Delta T. \qquad \text{Supplementary Equation (5)}$$

where R is reflectivity, T is temperature, and κ is the thermoreflectance coefficient. By linear fitting the relation $\Delta R/R$ and ΔT, κ can be extracted as shown in Supplementary Fig. 15.

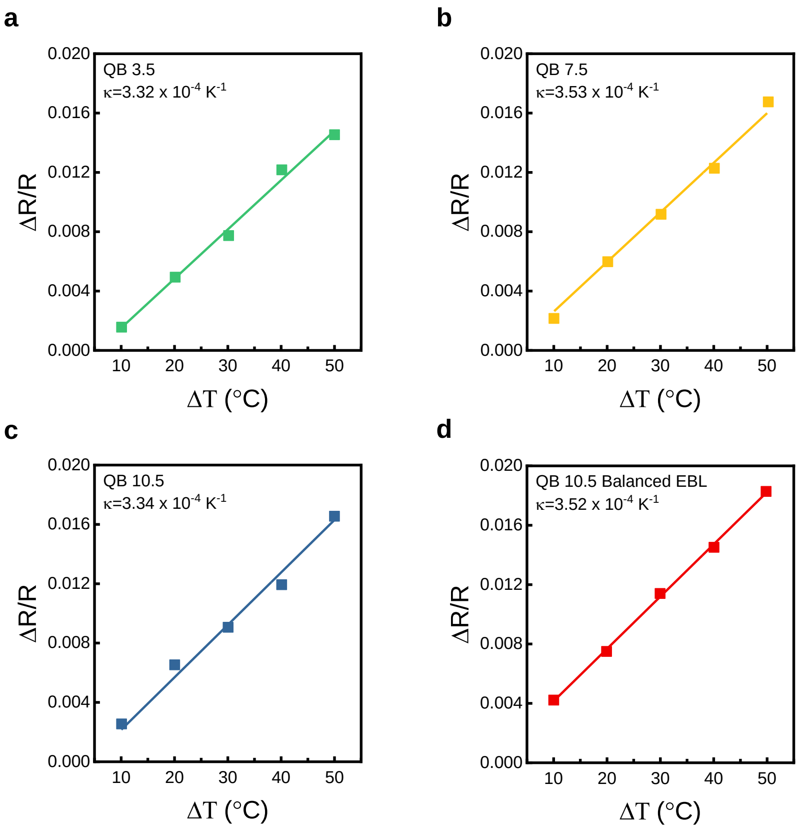

Supplementary Fig. 15 Linear fitting calibration results of thermoreflectance coefficient (κ) of different samples. a QB 3.5 **b** QB 7.5 **c** QB 10.5 **d** QB 10.5 Balanced EBL.

Supplementary References

[1] Lv, Q. et al. Realization of Highly Efficient InGaN Green LEDs with Sandwich-like Multiple Quantum Well Structure: Role of Enhanced Interwell Carrier Transport. *ACS Photonics* **6**, 130-138 (2019).

[2] Guan-Bo, L. et al. Effect of Quantum Barrier Thickness in the Multiple-Quantum-Well Active Region of GaInN/GaN Light-Emitting Diodes. *IEEE Photonics Journal* **5**, 1600207-1600207 (2013).

[3] Kim, S. et al. Fabrication of high-quality GaAs-based photodetector arrays on Si. *Applied Physics Letters* **110**, 153505 (2017).

[4] Hums, C. et al. Fabry-Perot effects in In Ga N⁄ Ga N heterostructures on Si-substrate. *Journal of Applied Physics* **101**, 033113 (2007).

[5] Ley, R. T. et al. Revealing the importance of light extraction efficiency in InGaN/GaN microLEDs via chemical treatment and dielectric passivation. *Applied Physics Letters* **116**, 251104 (2020).

[6] Smith, J. M. et al. Comparison of size-dependent characteristics of blue and green InGaN microLEDs down to 1 μm in diameter. *Applied Physics Letters* **116**, 071102 (2020).

[7] Wong, M. S. et al. Size-independent peak efficiency of III-nitride micro-light-emitting-diodes using chemical treatment and sidewall passivation. *Applied Physics Express* **12**, 097004 (2019).

[8] Olivier, F. et al. Influence of size-reduction on the performances of GaN-based micro-LEDs for display application. *Journal of luminescence* **191**, 112-116 (2017).

[9] Sheen, M. et al. Highly efficient blue InGaN nanoscale light-emitting diodes. *Nature* **608**, 56-61 (2022).

[10] Farzaneh, M. et al. CCD-based thermoreflectance microscopy: principles and applications. *Journal of Physics D: Applied Physics* **42**, 143001 (2009).
